# The efficacy of continuous nursing care for patients with chronic obstructive pulmonary disease: A randomized controlled trial protocol

**DOI:** 10.1097/MD.0000000000023974

**Published:** 2021-01-15

**Authors:** Xue Guo, Fengmin Men, Xingfen Han, Zhenying Wang

**Affiliations:** a Department of Respiratory and Critical Care Medicine; b Department of Internal Medicine; c Office of Health Care; d Respiratory Care Unit, Shandong Chest Hospital, Shandong, 250013, China.

**Keywords:** chronic obstructive pulmonary disease, continuous nursing care, protocol, quality of life

## Abstract

**Background:**

Chronic obstructive pulmonary disease (COPD) is a kind of disease that can be prevented and treated. It is characterized by the progressive limitation of airflow and is one of the most familiar human health barriers worldwide. For our program, the objective is to evaluate the impact of continuous care on the life quality of the COPD patients.

**Methods:**

This study will be implemented from June 2021 to March 2022 at Shandong Chest Hospital. The experiment was granted through the Research Ethics Committee of Shandong Chest Hospital (0029-4651). The criteria for inclusion involves:

(1) patients diagnosed with COPD;
(2) patients with expectorant, chronic cough, dyspnea, and other symptoms;
(3) patients who volunteered to take part in our study;
(4) have the cognitive ability to take part in interviews and fill in questionnaires.

The criteria for exclusion contains:

(1) patients with unstable physical conditions, for instance, congestive heart failure, recent myocardial infarction, acute cerebrovascular diseases, and malignant tumor;
(2) suffering from bronchial asthma or bronchiectasis;
(3) patients with serious physical dysfunction;
(4) patients who are unwilling to offer the informed consent to take part in this experiment.

For our research, the result measure is St. George's Respiratory Questionnaire (SGRQ).

**Results:**

Table 1 reflects the comparison results of 2 groups after the intervention.

**Conclusion:**

The continuous care on the basis of the theory of Information, Knowledge, Attitude, and Practice (IKAP) can promote the improvement of life quality in the COPD patients.

Trial registration number: researchregistry 6266.

## 1 Introduction

Chronic obstructive pulmonary disease (COPD) is a kind of disease that can be prevented and treated.^[1,2]^ It is characterized by the progressive limitation of airflow and is one of the most familiar human health barriers worldwide.^[3]^ While cardiovascular disease-related mortality has decreased evidently over the past 20 years, the number of COPD-related deaths has nearly doubled and COPD has become the fourth major cause of death in the world.^[4,5]^ In the US, it affects more than 15 million people and more than 210 million people worldwide.^[6]^ At the aim of better understanding and preventing the burden of COPD, the World Health Organization (WHO) has made major efforts of public health, but it forecasts that COPD disease will become the third most familiar cause of death globally by 2030.^[7]^ Bronchitis and emphysema can lead to a daily function loss and can be a growing and huge burden.^[8,9]^ The relevant treatment process includes repeated hospitalization, which brings high economic costs to the countries.

The theory of Information, Knowledge, Attitude, and Practice (IKAP) is a kind of relational theory, which has been utilized via a lot of scholars since the 1960s. The theoretical system primarily includes 4 continuous processes for conduction: the collection of information, knowledge acquisition, attitude formation, as well as the practice generation.^[10]^ The relationship between information, and knowledge, attitude as well as practice is gradual. The theory of IKAP has been utilized to the care of patients with a variety of diseases and has exhibited improved results. Nevertheless, it is rarely utilized to treat the COPD patients. Therefore, we conduct this randomized controlled trial protocol to evaluate the impact of continuous care on the life quality of the COPD patients.

## 2 Methods

This study will be implemented from June 2021 to March 2022 at Shandong Chest Hospital. The experiment was granted through the Research Ethics Committee of Shandong Chest Hospital (0029-4651) and recorded in research registry (researchregistry6266). Sequence of random numbers is generated by a computer. Sequentially numbered sealed opaque envelopes are used for the concealment of random numbers. All the patients taking part in our experiment are randomly divided to control or study group, and each group includes 30 patients.

### 2.1 Inclusion and exclusion criteria

The criteria for inclusion involves:

(1) patients diagnosed with COPD;
(2) patients with expectorant, chronic cough, dyspnea, and other symptoms;
(3) patients who volunteered to take part in our study;
(4) have the cognitive ability to take part in interviews and fill in questionnaires.

The criteria for exclusion contains:

(1) patients with unstable physical conditions, for instance, congestive heart failure, recent myocardial infarction, acute cerebrovascular diseases, and malignant tumor;
(2) suffering from bronchial asthma or bronchiectasis;
(3) patients with serious physical dysfunction;
(4) patients who are unwilling to offer the informed consent to take part in this experiment.

### 2.2 Nursing care management

In control group, all the patients will be given the routine nursing, and the education courses of COPD are conducted every 2 weeks. This contains the topics about the guidance on discharge process, the information on disease factors, lung rehabilitation, and the home oxygen therapy. In addition to routine care, intervention group patients are given continuous care based on the theory of IKAP. The nursing intervention primarily contains:

(1) Information collection: nurses collect the information on the psychological and health status of the patients.
(2) Knowledge acquisition: this phase includes the provision of the knowledge associated with healthcare to patients. Other topics contain trying to change the unhealthy lifestyles of patients and explaining the importance of respiratory exercises.
(3) Attitude generation: at this phase, the goal of nurses is to encourage all patients to remain positive. And nursing staff continues to study and consolidate the content of pre-discharge education of health. Nurses constantly evaluate the daily behavior and knowledge of patients, collect the data on the patients’ behavior change, and find out the major factors that hinder the behaviors change of patients.
(4) Practice formation: in this ultimate stage, the nurse guides patients to develop and then maintain good living habits and keep health. The care team of IKAP discusses a nursing plan with each patient conducts it. This includes the conduction of targeted personal interventions to help the patients form a proper understanding of the diseases, ensure that the patients have a capacity to conduct the programs of health education consciously, and facilitate the change to healthy behavior.

### 2.3 Outcomes

For our research, the result measure is the St. George's Respiratory Questionnaire (SGRQ).^[11]^ The questionnaire consists of 70 items divided into 3 dimensions, namely, symptoms, activities, and effects.^[12]^

### 2.4 Statistical analysis

Through utilizing the Microsoft Excel 2013, the data is recorded, and the data could be analyzed with IBM SPSS Statistics for Windows, version 20 (IBM Corp., Armonk, NY). And all data are represented via the proper characteristics, for instance, median, mean, and percentage. The categorical variables and continuous variables are respectively analyzed using independent *t* tests and *χ*^2^-tests. *P* value less than .05 indicates that there is statistical significance.

## 3 Result

Table 1 reflects the comparison results of 2 groups after the intervention.

**Table 1:** The comparison of patients in the 2 groups after intervention.

Variables	Control group (n = 30)	Study group (n = 30)	*P*
St. George's Respiratory Questionnaire Score			
Impact score			
Symptoms score			
Activity score			
Length of stay			
Complications			

## 4 Discussion

The dyspnea, low exercise tolerance, and mood disorders have formerly been demonstrated to affect the health status and life quality of patients with COPD.^[13–15]^ The theory of IKAP is beneficial to all these problems. The theoretical system of IKAP primarily includes 4 continuous processes for conduction, namely, the collection of information, knowledge acquisition, attitude formation, as well as the practice generation that encourage all the patients to abide by the plans of health education consciously and facilitate the changes of health behaviors.^[16]^ For this experiment, the innovations can be summed up in the use of the theory of IKAP in the continuous care of patients with COPD. Therefore, our research team has developed a set of planned, comprehensive, and continuous program of nursing intervention to help the patients understand the etiology, symptoms, and treatment in a real-time and comprehensive manner. This kind of intervention can enhance the confidence and attitude of patients to adhere to healthy behavior, and can guide the occurrence of behavior conducive to rehabilitation. The high-quality randomized controlled researches remains needed in-depth research.

## 5 Conclusion

The continuous care on the basis of the theory of IKAP can promote the improvement of life quality in the COPD patients.

## Author contributions

Zhenying Wang plans the study design. Fengmin Men reviews the protocol. Xingfen Han will collect data. Xue Guo writes the manuscript. All authors approve the submission.

**Conceptualization:** Fengmin Men.

**Data curation:** Fengmin Men.

**Funding acquisition:** Zhenying Wang.

**Investigation:** Xingfen Han.

**Methodology:** Xingfen Han.

**Writing – original draft:** Xue Guo.

## Abbreviations

Abbreviations: COPD = chronic obstructive pulmonary disease, IKAP = Information, Knowledge, Attitude, and Practice.
